# Milk-based culture of *Penicillium camemberti* and its component oleamide affect cognitive function in healthy elderly Japanese individuals: a multi-arm randomized, double-blind, placebo-controlled study

**DOI:** 10.3389/fnut.2024.1357920

**Published:** 2024-03-27

**Authors:** Mayuki Sasaki, Chisato Oba, Kentaro Nakamura, Hiroki Takeo, Hidemasa Toya, Keisuke Furuichi

**Affiliations:** ^1^Food Microbiology and Function Research Laboratories, R&D Division, Meiji Co., Ltd., Tokyo, Japan, Tokyo, Japan; ^2^Diastep Medical Corporation, Tokyo, Japan; ^3^HUMA R&D Corporation, Tokyo, Japan

**Keywords:** oleamide, *Penicillium camemberti*, brain-derived neurotrophic factor, cognitive function, working memory, elderly people, sleep, clinical trial

## Abstract

Dairy products and fermented foods have a reported association with maintained cognitive function. Camembert cheese, a dairy product fermented by the white mold *Penicillium camemberti*, has also been shown to enhance cognitive function *in vivo*. Oleamide, derived from the fermentation of the white mold, is a candidate for an active component, and expected to improve both cognitive function and sleep conditions. Thus, this study investigated whether the milk-based culture of white mold (MCW), and oleamide, could improve cognitive function and sleep state clinically. A multi-arm randomized, double-blind, placebo-controlled trial was conducted in Tokyo, Japan. 60 healthy Japanese individuals aged 50–75 who were aware of their cognitive decline were randomly and equally divided into three groups of 20 participants using computer-generated random numbers. Participants took either MCW (equivalent to 60 μg/day of oleamide), 60 μg/day of oleamide, or placebo capsules for 12 weeks. Serum BDNF, cognitive function by Cognitrax as primary and MCI Screen as secondary outcome, and sleep status using the Japanese version of the Pittsburgh Sleep Quality Index (PSQI-J) were assessed before and after intervention. The participants, outcome assessors and analysts, and research assistants were blinded to the group assignment. Of the 60 participants, 58 completed the study and were analyzed. No adverse events related to test foods were observed. The placebo group showed a negative rate of change in serum BDNF (−10.5% ± 19.7%), whereas the MCW and oleamide groups showed positive changes (2.0% ± 27.1% and 1.3% ± 13.5%, respectively). Cognitrax scores increased after 12 weeks in all groups. Conversely, the MPI score of the MCI Screen demonstrated a significant improvement in the MCW and oleamide groups compared to the placebo group (*p* = 0.013 and *p* < 0.001, respectively). The subscales, immediate free recall and delayed free recall, also significantly increased in them compared to the placebo group. Although PSQI-J revealed no significant differences among groups, the MCW and oleamide groups showed significant improvement after intervention in overall score, subjective sleep quality, and sleep latency. Our results suggest that MCW and its component, oleamide, are safe and contribute to maintaining cognitive functions, particularly short-term and working memory, and improving sleep state.

**Clinical trial registration**: https://center6.umin.ac.jp/cgi-open-bin/ctr/ctr_view.cgi?recptno=R000054792, identifier UMIN-CTR UMIN000048084.

## 1 Introduction

With ongoing advancements in science, technology, and medicine enhancing life expectancy, the global population is experiencing rapid aging. Conversely, healthy life expectancy has not elongated, and the health of older individuals has not improved from the previous generation, even in some regions of high-income countries (1). This is due to age-related declines in physical and cognitive functions. To prolong the healthy lifespan of the elderly, maintaining cognitive function along with physical function through daily life is essential (2).

The relationship between cognitive function and diet has been well-established. Examples include the consumption of n-3 polyunsaturated fatty acids such as docosahexaenoic acid (DHA) and eicosapentaenoic acid (EPA) (3–5), dietary fiber, and fermented foods (6, 7). Recent reports have also discussed the impact of dairy products on cognitive function. The Hisayama Cohort study in Japan indicates that consuming a high amount of milk and dairy products can lower the risk of dementia among the Japanese (8, 9). An epidemiological study in the United States has also showed a correlation between dairy product consumption and cognitive function (10). Cheese, a fermented dairy product, generates numerous bioactive compounds during ripening and may have health benefits (11). Kim et al. showed an inverse association between cheese consumption and lower cognitive function. These studies indicate that the intake of dairy and fermented dairy products may enhance cognitive function (12).

Nowadays, research specifically focusing on Camembert cheese and its components has also been conducted. Suzuki et al. carried out a clinical study involving elderly women with mild cognitive impairment, in which the participants consistently consumed either Camembert cheese or processed cheese over a three-month period (13). The study revealed that consuming Camembert cheese continuously for 3 months increased the levels of serum brain-derived neurotrophic factor (BDNF), a factor closely associated with cognitive function, compared to that of processed cheese. Ano et al. demonstrated through *in vivo* studies that Camembert cheese and its extracts can reduce the accumulation of amyloid-β, suppress the release of inflammatory cytokines, and enhance the production of hippocampal neurotrophic factor (14). Beta-lactopeptide, dehydroergosterol, and oleamide are potential components contributing to these effects (14–16). Above all, oleamide has been suggested to have physiological functions associated with cognitive function and sleep (17, 18). Oleamide naturally occurs in foods such as Camembert cheese, jujube (*Ziziphus jujuba*), and the essential oil of mountain celery seeds (19, 20). In Camembert cheese, it is considered that oleamide is produced through the amide bonding of ammonia and oleic acid derived from milk during the fermentation process by *P*. *camemberti* (14). Oleamide is reported to suppress inflammation and enhance microglial phagocytosis in the central nervous system (14). Additionally, administering oleamide to neonatal mice enhanced their learning and memory-related skills. Thus, oleamide is considered as one of the components in certain foods that contributes to cognitive enhancement (21). At the same time, oleamide is a lipid found from cerebrospinal fluids of sleep derived cats (22). It has been revealed that the intraperitoneal administration of oleamide in rats reduce sleep latency, slow-wave sleep, and motor activity, potentially enhancing sleep quality (17, 23). Thus, followed by additional findings, oleamide has long been described to have relationship with sleep state (24–26). Moreover, jujube fruit is also shown to prolong sleeping time and to decrease its locomotor activities, and although not stated in this review, oleamide contained in them could be one of the reasons of this effect (19). One possibility for the mechanism of these findings is that oleamide is an endogenous agonist of the cannabinoid receptor 1, in which its enhancement is known to be involved in cognition, motor function, memory, nociception, and sleep (27, 28). The efficacy of oleamide on cognitive function and sleep state has only been proven in animal experiments, and not in clinical trials.

We have developed a milk culture enriched with oleamide, using the white mold *P. camemberti*, based on the manufacturing method of Camembert cheese. To evaluate the impact of continuous intake of milk-based culture of white mold (MCW) and its active ingredient, oleamide, on cognitive function, a randomized, double-blind, placebo-controlled, parallel-group comparative study was carried out in healthy elderly individuals experiencing cognitive decline. Additionally, the impact of oleamide on sleep quality was assessed using patient-reported outcomes.

## 2 Methods

### 2.1 Ethical considerations

The study adhered to the Declaration of Helsinki, the Ethical Guidelines for Life Sciences and Medical Research Involving Human Subjects, and the Act on the Protection of Personal Information. The study was conducted after review and approval by the Yoga Allergy Clinic Clinical Research Ethics Review Committee (approval number: RD11002TS04). The study was pre-registered with the University Hospital Medical Information Network Clinical Trials Registry (registration number: UMIN000048084; registration on 17 June 2023).

### 2.2 Study participants

#### 2.2.1 Participants

Healthy Japanese adult men and women aged 50 to 75 years, who were aware of their cognitive decline, were recruited as volunteers. A total of 148 volunteers were briefed about the study details and provided written consent. After conducting screening tests, 60 participants who met all inclusion criteria and none of the exclusion criteria outlined in Table 1 were enrolled in the study. Under enrollment, people whose scores of MMSE are 22 or 23, who may be suspected of mild cognitive impairment, were judged as healthy by the principal doctor based on the results of the screening tests. The study collected data from participants at DiaStep Tokyo Skytree Ekimae Internal Medicine, Tokyo, Japan, between September and December 2022.

**Table 1:** Inclusion and exclusion criteria for this study.

**Inclusion criteria**	
(1)	Japanese men and women aged between 50 and 75 years old at the time of obtaining written consent.
(2)	Participant who is aware of their cognitive decline.
(3)	Participant who has a MMSE-J score of 22 points or more.
(4)	Participant who has received sufficient explanation of the purpose and content of the research, has the ability to consent.
**Exclusion criteria**	
(1)	Participant who has been diagnosed with dementia by a physician.
(2)	Participant who is taking medication or under medical treatment due to serious illness.
(3)	Participant who is under exercise therapy or dietetic therapy.
(4)	Participant who is at risk of showing allergic symptoms to 28 food allergens (7 allergens (egg, milk, wheat, peanut, shrimp, buckwheat, crab) required and 21 allergens (kiwifruit, walnut, soybean, banana, yam, cashew nut, peach, sesame, mackerel, salmon, squid, chicken, apple, matsutake mushroom, orange, beef, gelatin, pork, abalone, salmon roe, almond) recommended for labeling)
(5)	Participant who has or had a history of either drug or alcohol dependence syndrome.
(6)	Participant who currently attends a hospital for or has a history of mental disorder (depression, etc.) or sleep disorder (insomnia, sleep apnea syndrome, etc.)
(7)	Participant whose working hours are irregular due to night shifts, etc.
(8)	Participant who has extremely irregular lifestyle habits in terms of eating and sleeping.
(9)	Participant who has an extremely unbalanced diet.
(10)	Participant who smokes more than 21 cigarettes/day or drinks alcohol heavily (average net alcohol intake of about 60 g/day or more)
(11)	Participant with a serious current or previous illness such as brain disease, malignant tumor, immunological disease, diabetes, liver disease (hepatitis), renal disease, cardiac disease, thyroid disease, adrenal disease, or other metabolic disease.
(12)	Participant who takes supplements, health foods, etc. (including food for specified health uses and food with functional claims) or medicines that affect cognitive functions for 4 days or more per week.
(13)	Participant who has a habit of consuming cheese (an amount exceeding 7 pieces of 6P cheese/week or 7 slices of cheese/week).
(14)	Participant who has participated in other clinical studies within the past 3 months from the day of the consent acquisition or who is planning to participate in other clinical studies during the current study.
(15)	Participant who has participated in blood collection or donation of more than 200 mL within the past 1 month, or more than 400 mL within the past 3 months, from the day of the consent acquisition.
(16)	Participant who is planning to get pregnant after the day of informed consent or is currently pregnant or lactating.
(17)	Participant who has difficulty abiding to responding various survey forms.
(18)	Participant who is judged as an inappropriate candidate according to the screening data.
(19)	Participant who is considered as an inappropriate candidate by the doctor in charge.
(20)	Participant who is unable to cooperate with the countermeasures against SARS-CoV-2 infection and PCR testing as stipulated by the government and local authorities according to the status of SARS-CoV-2 infection.

#### 2.2.2 Determination of sample size

We referred to a three-arm clinical trial studying changes in cognitive function through consumption of various food components using Cognitrax, as there were no prior studies evaluating cognitive function with oleamide. In the study by Baba et al., significant results were obtained from testing 17 participants in each group (29). Assuming a potential 10–15% dropout rate from previous studies, we aimed to have 60 participants in total for this study, divided evenly across three groups to have 20 participants each.

### 2.3 Design of the study

The study was conducted as a multi-arm, randomized, double-blind, placebo-controlled, parallel-group comparative study. The study group allocator stratified and randomized 60 participants who were selected through a screening test (SCR) to oleamide, MCW, or placebo groups. Stratified randomization was conducted based on gender, age, and neurocognitive index (NCI) score from Cognitrax at SCR, aiming for equal distribution among groups. The similarity of conditions between groups after distribution was ensured based on computer-generated random numbers.

The study group allocator, independent from the contract research organization and research institute, maintained seal and strictly kept of the food randomization list until unblinding. The codebreaking was conducted after all data were finalized. The blinding was properly maintained for all parties and study participants, except for the study group allocator. The test foods were consumed for 12 weeks, with evaluations conducted before and after this 12-week period. On the day of the post-intervention test, study participants fasted for 6 h before undergoing various tests; urinalysis, vital signs and physical measurements, the Japanese version of the Pittsburgh Sleep Quality Index (PSQI-J), blood tests (including blood biochemistry, hematology, BDNF), the Japanese version of the MCI Screen, and Cognitrax. The Cognitrax test was conducted only at SCR and after intervention.

### 2.4 Intervention

The MCW group was given capsules containing 300 mg of lyophilized powder of milk-based culture of white mold *P. camemberti* (MCW). On the other hand, the oleamide group received capsules containing oleamide (Nootropics Depot, United States). Both capsules were formulated to administrate equivalent doses of 60 μg oleamide per serving. The placebo capsules primarily contained cellulose, without oleamide or MCW. Detailed composition of each test food is shown in Table 2. The study participants were given test foods each month, packaged in identical soft capsules that were indistinguishable in color, odor, and flavor, and sealed in aluminum bags. The test food was to be taken as four capsules daily after the same meal of the day, and taken with either cold or lukewarm water, until the day before the post-intervention test. If participants forgot to take the test food after the pre-determined meal and realized the mistake during the same day, they were allowed to take it on that day only. Research assistants supervised the consumption of test foods through the lifestyle questionnaire that the participants were asked to fill out daily. If the test food had not been consumed, the research assistants made a phone call to the participant to ask the reason and to remind them to consume it every day.

**Table 2:** Composition of test foods in this study.

Ingredient	Composition rate (%)		
	OAD	MCW	Placebo
Oleamide	0.010	0.000	0.000
Powdered MCW	0.000	48.891	0.000
Excipients	71.490	24.442	71.500
Coloring/flavoring	1.833	0.000	1.833
Capsule	26.667	26.667	26.667
Total (%)	100.000	100.000	100.000

MCW, milk-based culture of white mold, which is produced by fermenting a mixture of milk protein concentrate and butter with *Penicillium camemberti*.

### 2.5 Outcomes

#### 2.5.1 Primary outcomes

Serum BDNF and Cognitrax were chosen as the primary outcomes. Measurement of serum BDNF concentrations was contracted to Healthcare Systems Co., Ltd. (Aichi, Japan), using sandwich enzyme-linked immunosorbent assay (ELISA) kits for BDNF (DuoSet; R&D Systems, Minneapolis, MN, United States), and was performed according to the manufacturer’s protocol. Blood samples for BDNF were collected in the morning from 10:00 to 10:30 on the day of the pre-intervention test and on the day of post-intervention test at 12 weeks. The BDNF assays were conducted using the same batch of kits.

Cognitrax is an online cognitive function assessment test using computer. It provides a comprehensive evaluation of various domains including memory, attention, processing speed, and executive function (30). Cognitrax results are normalized according to age and educational level and are evaluated based on 12 different indices: Neurocognition Index (NCI), Composite Memory, Verbal Memory, Visual Memory, Psychomotor Speed, Reaction Time, Complex Attention, Cognitive Flexibility, Processing Speed, Executive Functioning, Simple Visual Attention, and Motor Speed. Cognitrax was assessed at SCR and the post-intervention test.

#### 2.5.2 Secondary outcomes

The secondary outcomes were determined using the Japanese version of the MCI Screen and PSQI-J. The MCI Screen is a cognitive function test that accurately differentiates normal cognitive function and mild cognitive impairment (MCI) or mild dementia. The MCI Screen comprises the following three stages: (1) The assessor recites 10 words, and the participant immediately repeats them. This process is repeated three times; (2) The assessor asks 10 questions where the participant shall identify the odd one out among three animals; and (3) The participant repeats the words from step (1) that they can remember without the assessor reciting any. Based on the results of (1) to (3), Z-scores were calculated for both immediate and delayed free recall. Additionally, an overall index, the Memory Performance Index (MPI) score ranging from 0 to 100, was derived using demographic data such as gender, age, and years of learning experience. In this research, we utilized the Japanese version of the MCI Screen, which is validated in Japanese (31).

PSQI-J was utilized to evaluate sleep state (32, 33). Participants were to grade their average subjective sleep state over the past month based on seven factors: sleep quality, sleep latency, sleep duration, habitual sleep efficiency, sleep disturbance, use of sleep medication, and daytime dysfunction. The total score was used to determine PSQI-J.

The MCI Screen and PSQI-J were carried out twice, at the pre-intervention test and the post-intervention test.

#### 2.5.3 Safety evaluation

Safety was assessed through vital signs (systolic and diastolic blood pressure and pulse rate), physical measurements (body weight and body mass index), and blood biochemical tests [triglyceride (TG), total cholesterol (T-Cho), blood urea nitrogen (BUN), total bilirubin (T-Bil), total protein (TP), albumin (Alb), γ-glutamyl transpeptidase (γ-GTP), aspartate aminotransferase (AST), alanine aminotransferase (ALT), creatinine (Cr), uric acid (UA), LDL-cholesterol (LDL-Cho), blood glucose, HDL-cholesterol (HDL-Cho), alkaline phosphatase (ALP), lactate dehydrogenase (LDH), hemoglobin A1c (HbA1c)], hematological tests [white blood cell (WBC), red blood cell (RBC), hemoglobin (Hb), hematocrit (Ht), mean corpuscular volume (MCV), mean corpuscular hemoglobin (MCH), mean corpuscular hemoglobin concentration (MCHC), platelet (PLT)], urinalysis (pH, specific gravity, protein qualitative, glucose qualitative, urobilinogen, occult blood reaction, bilirubin, ketone bodies) and adverse events. These parameters were measured thrice: during SCR, pre-intervention test, and post-intervention test. To account for diurnal variation, blood samples for the pre-intervention test and post-intervention test were collected in the morning from 10:00 to 10:30.

### 2.6 Statistical analysis

This study analyzed the Per-Protocol Set (PPS) population. The Dunnett test was used to compare the BDNF, Cognitrax, and MCI Screen among groups, while the paired t-test was used for comparison between before and after the intake of test foods. PSQI-J results were compared among different groups using the Steel test, and these results were further compared with pre-intervention results using the Wilcoxon signed rank test. The backgrounds of the study participants were compared using analysis of variance. Safety endpoints were analyzed among groups using Dunnett, Steel, and Fisher’s exact probability tests, and compared to pre-intervention test results using paired t-test and Wilcoxon signed-rank test, based on specific data characteristics. The significance level for all tests was 5% two-sided, and statistical analysis was performed using statistical analysis software (IBM^Ⓡ^: SPSS^Ⓡ^ Statistics 27 and EZR version 1.55). The mean, standard deviation, and 95% confidence interval are displayed for the participant background and efficacy endpoints (excluding PSQI-J), and interquartile range are displayed for PSQI-J.

## 3 Results

### 3.1 Characteristics of the study participants

**Figure 1:**
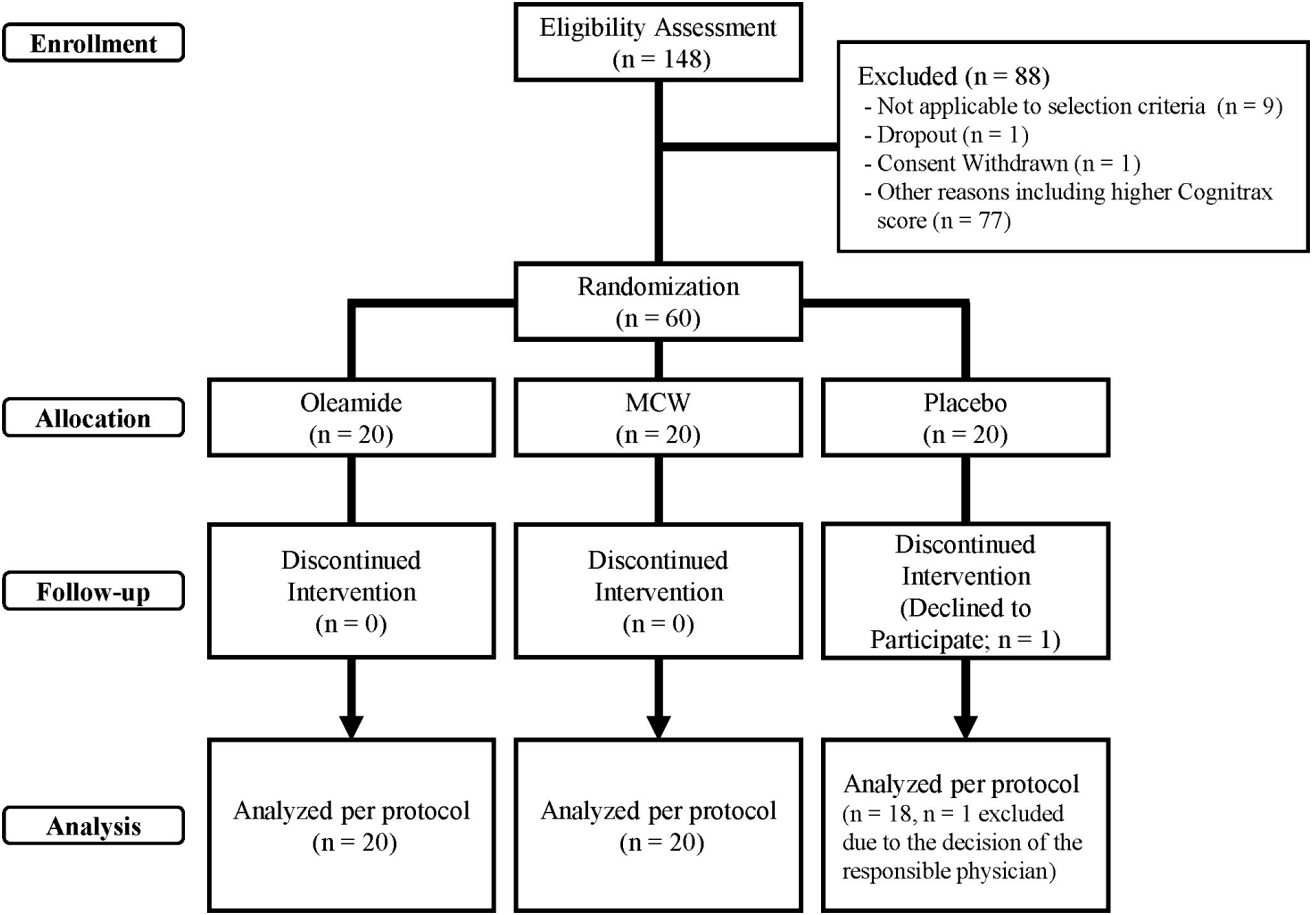
CONSORT flow diagram of study participants. MCW, Milk-Based Culture of White Mold.

The disposition of study participants is shown in Figure 1. From June to July 2022, 148 individuals were recruited and screened for the study. 60 healthy male and female participants were enrolled in this study, and randomly allocated evenly across three groups of 20 participants: MCW, oleamide, and placebo.

During the study, one participant in the placebo group withdrew due to personal reasons, leaving a total of 59 participants (20 in the MCW group, 20 in the oleamide group, and 19 in the placebo group) by the end of the study. After intervention, one placebo participant was excluded due to a protocol violation involving medication that could affect cognitive ability. Consequently, 58 participants remained in the PPS: 20 in the MCW group, 20 in the oleamide group, and 18 in the placebo group. However, Cognitrax results of two participants, one from the oleamide group and the other in the placebo group, were excluded from the PPS analysis due to reliability concerns: operational error of the computer by one participant and partial non-calculation of the results for the other.

The demographic characteristics of the participants at baseline are summarized in Table 3. There were no significant differences between the placebo group and the other two groups.

**Table 3:** Characteristics of participants.

	All			Oleamide			MCW			Placebo			*p*-value
Number	58			20			20			18			-
**Gender**													
Male	32 (55.2%)			11 (55.0%)			11 (55.0%)			10 (55.6%)			1.00
Female	26 (44.8%)			9 (45.0%)			9 (45.0%)			8 (44.4%)			
Age (years)	62.6	±	8.0	62.3	±	9.1	62.8	±	7.3	62.8	±	8.0	0.97
Height (cm)	162.7	±	9.7	162.8	±	10.1	163.1	±	8.7	162.2	±	10.9	0.96
Body weight (kg)	60.7	±	11.4	60.3	±	10.2	60.9	±	12.1	60.9	±	12.6	0.98
BMI (kg/m^2^)	22.7	±	2.8	22.6	±	2.7	22.7	±	3.3	22.9	±	2.6	0.97
MMSE-J (points)	26.5	±	1.9	26.7	±	2.2	26.6	±	1.9	26.4	±	1.6	0.91
NCI (points)	96.1	±	9.1	96.6	±	8.7	96.1	±	8.0	95.6	±	10.9	0.95
Compliance rate of test food intake	100.0	±	0.2	99.9	±	0.4	100.0	±	0.0	100.0	±	0.0	0.14

MCW, Milk-Based Culture of White Mold; BMI, Body Mass Index; MMSE-J, Japanese Mini Mental State Examination; NCI, Cognitrax Neurocognition Index. Data are presented as mean ± standard deviation for all groups. Analysis was conducted using ANOVA.

### 3.2 Primary outcomes

#### 3.2.1 BDNF

**Figure 2:**
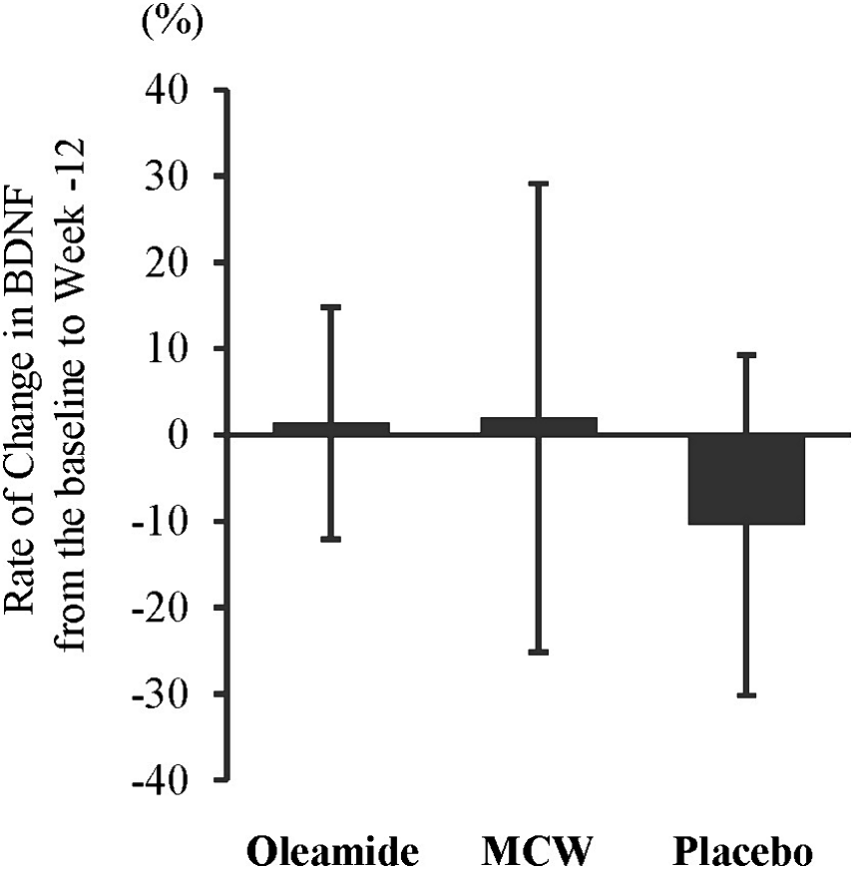
Rate of Change in BDNF from the baseline to week-12. The bars represent the average rate of change, while the error bars denote the standard deviation. MCW, Milk-Based Culture of White Mold.

At 12 weeks, the oleamide group had significantly higher BDNF levels than those of the placebo group (Table 4, *p* = 0.005). The MCW and oleamide groups exhibited a positive rate of change in BDNF levels, whereas a negative rate of change was observed in the placebo group. However, no significant differences in the rate of change were found among the groups (Table 4; Figure 2).

**Table 4:** Comparison of serum BDNF.

	Group	*N*	Baseline			Week-12			Change rate (%)		
			Mean ± SD	95% CI	*p*-value	Mean ± SD	95% CI	*p*-value	Mean ± SD	95% CI	*p*-value
BDNF (ng/mL)	Oleamide	20	41.6 ± 11.6	(−2.6–11.5)	0.26	41.3 ± 9.4	(2.3–14.4)	0.005^**^	1.3 ± 13.5	(−3.6–27.2)	0.15
	MCW	20	35.4 ± 7.9	(−8.8–5.3)	0.79	34.5 ± 4.8	(−4.6–7.5)	0.80	2.0 ± 27.1	(−3.0–27.8)	0.13
	Placebo	18	37.2 ± 8.6				33.0 ± 9.7			−10.5 ± 19.7	

BDNF, Brain-Derived Neurotrophic Factor; MCW, Milk-Based Culture of White Mold. Data are presented as mean ± standard deviation (SD) for all groups. ^*^p < 0.05, ^**^p < 0.01, analyzed by Dunnett’s test (vs. placebo).

#### 3.2.2 Cognitrax

After 12 weeks of intake, all parameters improved in all groups, except for Simple Visual Attention in the MCW group, as compared to baseline (Table 5). However, there were no significant differences at 12 weeks among the groups (Table 5).

**Table 5:** Comparison of standardized scores on Cognitrax.

	Group	*N*	Baseline			Week-12			Change rate (%)		
			Mean ± SD	95% CI	*p*-value	Mean ± SD	95% CI	*p*-value	Mean ± SD	95% CI	*p*-value
Neurocognition index (NCI)	Oleamide	19	97.5 ± 8.0	(−4.7–9.1)	0.69	101.6 ± 6.5	(−7.3–5.2)	0.89	4.1 ± 7.0	(−8.6–2.1)	0.29
	MCW	20	96.1 ± 8.0	(−6.0–7.6)	0.95	102.3 ± 8.9	(−6.6–5.8)	0.98	6.2 ± 7.7	(−6.5–4.1)	0.82
	Placebo	17	95.3 ± 11.2			102.6 ± 9.2			7.4 ± 6.5		
Composite memory	Oleamide	19	94.9 ± 15.3	(−6.8–18.6)	0.47	102.3 ± 13.6	(−11.5–9.8)	0.98	7.4 ± 16.2	(−18.6–5.1)	0.33
	MCW	20	95.3 ± 15.2	(−6.2–18.8)	0.41	101.3 ± 11.4	(−12.4–8.6)	0.89	6.0 ± 12.5	(−19.8–3.5)	0.20
	Placebo	17	89.0 ± 19.7			103.1 ± 17.0			14.1 ± 18.1		
Verbal memory	Oleamide	19	92.5 ± 19.0	(−15.1–14.7)	1.00	101.1 ± 14.6	(−13.6–6.7)	0.65	8.5 ± 16.0	(−16.2–9.7)	0.78
	MCW	20	95.0 ± 17.8	(−12.5–17.0)	0.92	101.6 ± 10.3	(−13.0–7.0)	0.72	6.6 ± 15.9	(−18.0–7.6)	0.55
	Placebo	17	92.7 ± 22.4			104.5 ± 15.1			11.8 ± 19.5		
Visual memory	Oleamide	19	99.5 ± 14.5	(−2.3–21.7)	0.13	102.8 ± 13.5	(−9.2–13.0)	0.89	3.4 ± 21.9	(−22.0–6.4)	0.35
	MCW	20	98.3 ± 14.5	(−3.4–20.3)	0.19	100.9 ± 14.2	(−11.0–11.0)	1.00	2.7 ± 13.8	(−22.5–5.5)	0.29
	Placebo	17	89.8 ± 18.6			100.9 ± 16.5			11.2 ± 19.8		
Psychomotor speed	Oleamide	19	101.0 ± 12.4	(−15.1–3.9)	0.31	105.9 ± 10.6	(−12.2–3.1)	0.31	4.9 ± 8.8	(−5.4–7.6)	0.90
	MCW	20	103.7 ± 12.0	(−12.3–6.5)	0.71	110.2 ± 9.2	(−7.8–7.3)	1.00	6.5 ± 9.0	(−3.8–9.0)	0.55
	Placebo	17	106.6 ± 13.3			110.4 ± 10.5			3.8 ± 7.7		
Reaction time	Oleamide	19	93.1 ± 12.9	(−3.2–18.0)	0.20	94.6 ± 10.9	(−7.4–9.6)	0.94	1.6 ± 9.5	(−15.3–2.7)	0.20
	MCW	20	85.9 ± 13.7	(−10.3–10.7)	1.00	92.6 ± 10.3	(−9.4–7.4)	0.95	6.7 ± 14.7	(−10.1–7.8)	0.94
	Placebo	17	85.6 ± 15.5			93.5 ± 12.5			7.9 ± 10.6		
Complex attention	Oleamide	19	100.3 ± 14.7	(−11.9–9.3)	0.95	103.7 ± 11.9	(−11.5–7.1)	0.81	3.4 ± 13.3	(−10.3–8.5)	0.96
	MCW	20	102.2 ± 11.9	(−9.9–11.1)	0.99	106.4 ± 12.4	(−8.7–9.7)	0.99	4.2 ± 12.3	(−9.4–9.1)	1.00
	Placebo	17	101.6 ± 15.5			105.9 ± 12.7			4.3 ± 11.5		
Cognitive flexibility	Oleamide	19	98.0 ± 10.3	(−4.4–12.8)	0.44	101.2 ± 10.6	(−8.5–9.8)	0.98	3.2 ± 10.3	(−11.1–4.0)	0.46
	MCW	20	94.1 ± 11.3	(−8.2–8.7)	1.00	101.1 ± 14.9	(−8.5–9.6)	0.99	7.0 ± 11.9	(−7.2–7.7)	0.99
	Placebo	17	93.8 ± 12.4			100.5 ± 9.6			6.7 ± 6.4		
Processing speed	Oleamide	19	112.1 ± 11.3	(−10.8–10.8)	1.00	115.7 ± 14.2	(−10.5–8.9)	0.97	3.6 ± 8.5	(−7.6–6.0)	0.95
	MCW	20	114.3 ± 13.9	(−8.5–12.8)	0.86	119.9 ± 11.1	(−6.3–12.9)	0.64	5.6 ± 8.0	(−5.5–7.9)	0.89
	Placebo	17	112.1 ± 17.2			116.5 ± 12.9			4.4 ± 10.6		
Executive function	Oleamide	19	98.3 ± 9.6	(−3.7–13.0)	0.35	101.5 ± 10.9	(−7.9–10.4)	0.93	3.3 ± 10.0	(−10.8–4.0)	0.47
	MCW	20	94.4 ± 11.7	(−7.5–9.1)	0.96	101.1 ± 15.2	(−8.2–9.9)	0.97	6.7 ± 11.8	(−7.3–7.3)	1.00
	Placebo	17	93.6 ± 11.7			100.3 ± 8.8			6.7 ± 6.1		
Simple visual attention	Oleamide	19	92.7 ± 24.3	(−21.6–7.5)	0.44	101.7 ± 15.0	(−9.9–10.9)	0.99	9.0 ± 24.2	(−9.1–24.2)	0.48
	MCW	20	103.4 ± 9.8	(−10.8–17.9)	0.80	101.2 ± 8.4	(−10.3–10.2)	1.00	−2.2 ± 14.0	(−20.0–12.9)	0.84
	Placebo	17	99.8 ± 21.1			101.2 ± 16.9			1.4 ± 26.6		
Motor speed	Oleamide	19	94.1 ± 13.7	(−17.0–3.1)	0.21	97.5 ± 10.4	(−14.0–2.4)	0.19	3.4 ± 9.2	(−5.9–8.2)	0.91
	MCW	20	95.3 ± 13.8	(−15.6–4.1)	0.32	100.7 ± 12.1	(−10.7–5.5)	0.68	5.5 ± 10.6	(−3.8–10.1)	0.49
	Placebo	17	101.0 ± 11.9			103.3 ± 9.4			2.3 ± 7.8		

MCW, Milk-Based Culture of White Mold. Data are presented as mean ± standard deviation (SD) for all groups. Analyzed by Dunnett’s test (vs. placebo).

### 3.3 Secondary outcomes

#### 3.3.1 MCI screen

**Figure 3:**
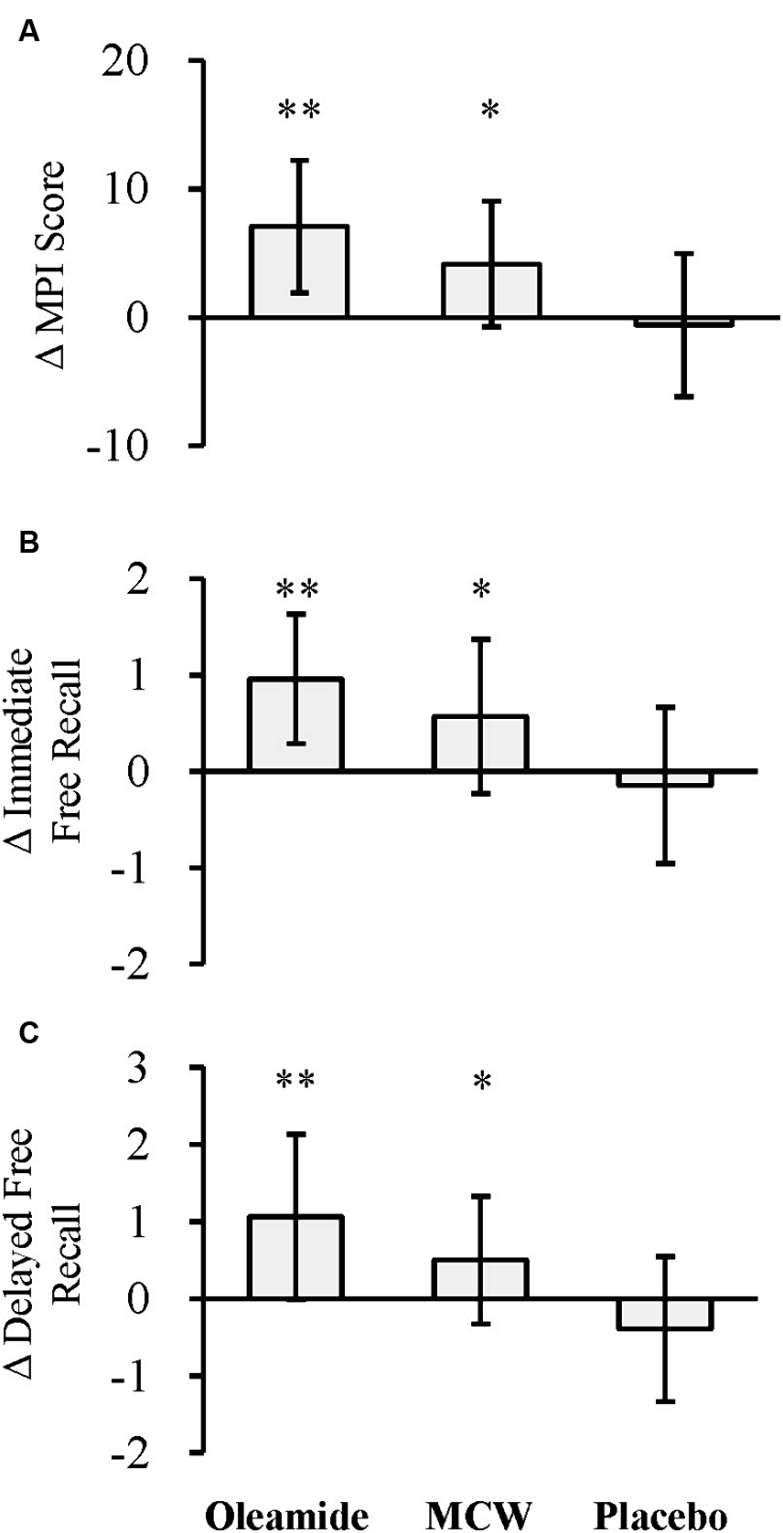
Changes in MCI Screen. **(A)** Δ MPI score, **(B)** Δ immediate free recall, and **(C)** Δ delayed free recall. The bars represent the mean, while the error bars denote the standard deviation. ^*^p < 0.05, ^**^p < 0.01, analyzed by Dunnett’s test (vs. placebo). MCW, Milk-Based Culture of White Mold; MPI, Memory Performance Index.

The changes of the MPI score at 12 weeks (Δ MPI score) was significantly greater in the MCW and oleamide groups compared to the placebo group (Figure 3A; Table 6; *p* = 0.013 in the MCW group and *p* < 0.001 in the oleamide group).

**Table 6:** Comparison of MCI screen scores.

	Group	*N*	Baseline			Week-12			Δ		
			Mean ± SD	95% CI	*p*-value	Mean ± SD	95% CI	*p*-value	Mean ± SD	95% CI	*p*-value
MPI score	Oleamide	20	57.78 ± 8.53	(−8.86–3.16)	0.455	64.86 ± 8.07	(−1.09–10.72)	0.123	7.08 ± 5.16	(3.83–11.49)	<0.001^**^
	MCW	20	57.43 ± 8.32	(−9.20–2.82)	0.379	61.59 ± 8.13	(−4.36–7.45)	0.775	4.15 ± 4.90	(0.91–8.57)	0.013^*^
	Placebo	18	60.63 ± 7.54			60.04 ± 7.84			−0.58 ± 5.57		
Immediate free recall	Oleamide	20	−1.47 ± 1.04	(−1.38–0.29)	0.241	−0.51 ± 1.00	(−0.23–1.35)	0.198	0.96 ± 0.67	(0.54–1.67)	<0.001^**^
	MCW	20	−1.47 ± 1.23	(−1.38 – 0.29)	0.241	−0.90 ± 1.18	(−0.62–0.96)	0.845	0.57 ± 0.80	(0.15–1.28)	0.010^*^
	Placebo	18	−0.92 ± 1.13			−1.07 ± 1.02			−0.14 ± 0.81		
Delayed free recall	Oleamide	20	−1.22 ± 0.97	(−1.30–0.24)	0.210	−0.15 ± 0.97	(0.20–1.65)	0.010^*^	1.07 ± 1.07	(0.76–2.16)	<0.001^**^
	MCW	20	−1.14 ± 1.02	(−1.23–0.31)	0.305	−0.64 ± 0.86	(−0.29–1.16)	0.295	0.50 ± 0.83	(0.19–1.60)	0.010^*^
	Placebo	18	−0.68 ± 1.15			−1.08 ± 1.13			−0.39 ± 0.94		

MCW, Milk-Based Culture of White Mold; MPI, Memory Performance Index; CI, Confidence Interval. Data are presented as the mean ± standard deviation (SD). ^*^p < 0.05, ^**^p < 0.01, analyzed by Dunnett’s test (vs. placebo).

The changes of the Z-scores for immediate and delayed free recall (Δ Immediate free recall and Δ Delayed free recall, respectively) were also significantly greater in the MCW and oleamide groups compared to the placebo group (Figure 3B; Table 6: immediate free recall; *p* = 0.010 in the MCW group and *p* < 0.001 in the oleamide group; Figure 3C; Table 6: delayed free recall; *p* = 0.010 in the MCW group and p < 0.001 in the oleamide group).

#### 3.3.2 PSQI-J

**Figure 4:**
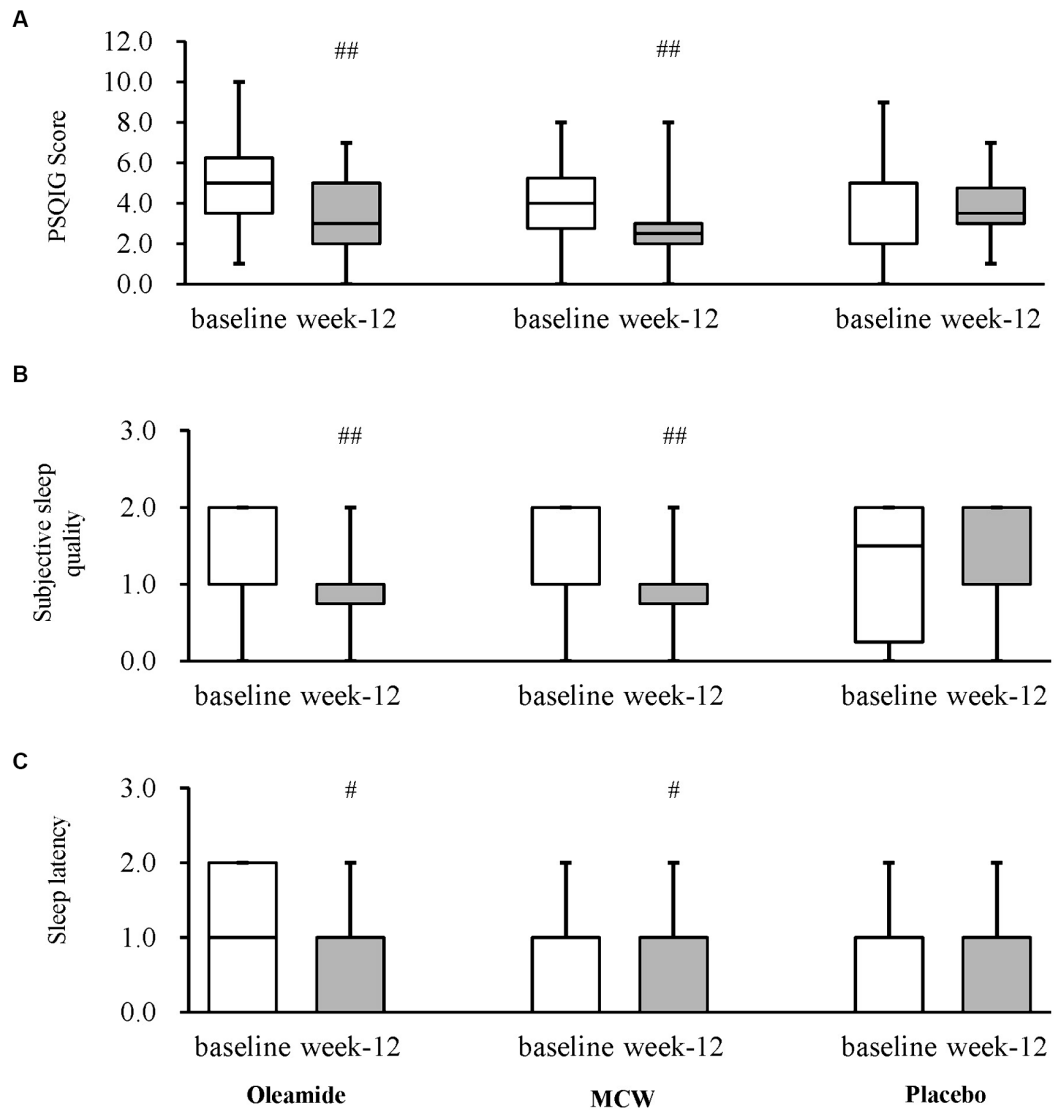
Changes in PSQIG Score. **(A)** PSQIG Score, **(B)** Subjective sleep quality, and **(C)** Sleep latency. Box plot indicates interquartile range (IQR). White boxes show the participants’ state at baselines and gray ones show their state at week-12. ^#^*p* < 0.05, ^##^*p* < 0.01, performed by Wilcoxon signed rank test (vs. baseline). Oleamide and MCW groups showed no significance compared to placebo group (analyzed by Steel’s test). PSQIG, Pittsburgh Sleep Quality Index Global score; MCW, Milk-Based Culture of White Mold.

After 12 weeks of consumption, both the MCW and oleamide groups showed significantly lower overall scores than baseline (*p* = 0.002 in the MCW group and *p* = 0.003 in the oleamide group). Conversely, the placebo group exhibited no significant changes (Figure 4A; Table 7). Furthermore, the MCW group and the oleamide group both showed significant improvement in sleep quality and sleep latency scores after intervention (*p* < 0.001, *p* = 0.033 in the MCW group and *p* < 0.001, *p* = 0.014 in the oleamide group, respectively; Figures 4B,C; Table 7). However, no significant differences were observed among the groups at 12 weeks (Figure 4; Table 7).

**Table 7:** Comparison of PSQI-J scores.

Variant	Group	*N*	Baseline median (25–75 percentile)		*p*-value	Week-12 Median (25–75 percentile)		*p*-value (between groups)	*p*-value (within group)
PSQIG	Oleamide	20	5.0	(3.5–6.3)	0.54	3.0	(2.0–5.0)	0.91	^**^0.003
	MCW	20	4.0	(2.8–5.3)	1.00	2.5	(2.0–3.0)	0.06	^**^0.002
	Placebo	18	5.0	(2.0–5.0)		3.5	(3.0–4.8)	-	0.41

Pittsburgh Sleep Quality Index Global score; MCW, Milk-based Culture of White Mold; Data are represented as the median and the interquartile range. **p < 0.01, analyzed by Wilcoxon’s signed rank test.

### 3.4 Safety evaluations

After 12 weeks of intervention, vital signs, physical measurements, blood parameters, and urinalysis were within normal range, although there were significant differences in some parameters compared to the placebo group (Supplementary Tables 1–5). During the study period, four participants experienced seven adverse events, all of which were deemed by the investigators to be unrelated to the test foods. The incidence of adverse events did not significantly differ among the three groups (*p* = 0.532). In conclusion, the safety of the test foods was confirmed over a 12-week intervention period.

## 4 Discussion

In this randomized, double-blind, placebo-controlled study, we examined the effects of MCW and its potential active ingredient, oleamide, on cognitive function in healthy older Japanese individuals experiencing subjective cognitive decline. The results demonstrated that both 60 μg of oleamide and MCW containing 60 μg of oleamide, which were administered for 12 weeks, significantly improved the MPI score of the MCI Screen compared to placebo, indicating a positive effect on cognitive function. Additionally, both the MCW and oleamide groups demonstrated significant improvement in immediate and delayed free recall scores, a sub-category of the MCI Screen, compared to the placebo group. Even though the primary outcome, which was set as Cognitrax, was not achieved in this study, these findings well indicate that continuous consumption of oleamide effectively preserves cognitive abilities, particularly working and short-term memory, in older individuals who are aware of their cognitive decline. This is the first study to directly present the efficacy of oleamide on cognitive function in a clinical trial. Short-term and working memory process verbal, audio, visual, and spatial information encountered in our daily and social interactions. Generally, aging leads to a decline in short-term and working memory (34). Furthermore, it has been extensively debated that enhancing and maintaining short-term and working memory functions could potentially inhibit the onset and progression of dementia (35). This study suggests that intake of oleamide or oleamide-rich foods could potentially prevent dementia by preserving short-term and working memory functions.

Results from our research presented that while the MCI Screen demonstrated significant improvement, the Cognitrax did not show a noticeable difference compared to the placebo (Figure 3; Table 5). This contradiction may be attributed to the different target populations of the two tests. The Cognitrax test is designed for a broad spectrum of individuals, ranging from people with normal cognitive function to patients with dementia (30). The MCI Screen is designed to evaluate cognitive function in healthy individuals and individuals with mild cognitive impairment (MCI) (36). It can distinguish between healthy individuals and individuals with MCI with an accuracy of 97%–99% (31). Given this discrepancy, it is inferred that the MCI Screen is more sensitive to individuals with near-normal cognitive function than the Cognitrax. Indeed, this study involved elderly participants aware of their cognitive decline. However, their baseline values for both tests were high (Cognitrax: mean 90–100; Table 5, MCI Screen: mean 57–60; Figure 3; Table 6) in contrast to our prediction, indicating that their cognitive functions were relatively well-maintained. Therefore, in this study, significant differences were only observed in the MCI Screen, which we infer to be highly sensitive to individuals with near-normal cognitive function. Apart from our research, there have been clinical studies in healthy participants that detected the efficacy of foods on cognitive function. These effects are more sensitively detected by the MCI Screen compared to Cognitrax, as the MCI Screen aligns more closely with the original test target. A clinical study evaluating supplements containing propolis extract, curcumin, and other substances found significant differences in the MCI Screen results, but not in Cognitrax (37). According to our current research and the previous study (37), the MCI Screen may have had been more effective than Cognitrax in detecting changes in cognitive function in healthy participants in human clinical studies like this trial. Also, neither the mechanism-based difference between the two assessments nor the mode of action of oleamide has been revealed to this day. The aspects mentioned above will be of concern in the future studies.

In this study, the oleamide group showed a significantly higher serum BDNF level than that of the placebo group after 12 weeks of intake. Moreover, the placebo group exhibited a negative rate of change, whereas both the oleamide and MCW groups displayed a positive rate of change, with no regression to the mean observed. Previous reports suggest an association between serum BDNF levels and cognitive function (38). Suzuki et al. demonstrated that the continuous intake of Camembert cheese raises serum BDNF levels (13). They partially attributed this association to the presence of oleamide in Camembert cheese (13). Our result is consistent with this prior study, suggesting that consuming foods containing oleamide could enhance cognitive function through BDNF elevation. Nevertheless, the increase of serum BDNF level in the MCW group was insignificant despite the amount of oleamide ingested. The reason for this result is two-fold: relatively high baseline BDNF level in the oleamide group compared to the MCW group and significant decrease in blood BDNF levels in the placebo group at 12 weeks. Additionally, the current study revealed that the intake of MCW and oleamide exhibited similar changes not only in serum BDNF levels but also in the MCI Screen. Generally, consuming mixtures of different elements like extracts or fermented products may not yield the same effects as consuming a single component. The current findings indicate that MCW and oleamide exhibit similar efficacy on human cognitive function.

The efficacy of oleamide consumption on sleep quality was also assessed in this study. Both the MCW and oleamide groups demonstrated significant improvement from baseline, although not significant when compared to the placebo group. While the potential efficacy of oleamide intake on sleep has been demonstrated in rats (17, 23), this is the first study to report its effect on human sleep. Although further evidence needs to be accumulated focusing on sleep quality, this study indicates that the consumption of oleamide may have a beneficial effect on sleep in humans as well.

While MCW and oleamide both showed their contribution to the improvement on cognitive abilities and sleep state, it is fairly possible that agents other than oleamide have also contributed to the improvement of the participants’ cognitive abilities or sleep states. In fact, MCW did seem to be more effective than oleamide concerning PSQI-J scores. On the other hand, when we look at the results shown in Figures 1–4, it could be observed that the extent of their improvement on each measure is fairly the same when comparing MCW and oleamide alone. Therefore, while we cannot completely defy the contribution of other components, we consider that oleamide is the main active constituent.

One limitation of this study is that the demographics of the participants were minimal. We have only tested on healthy individuals whose ages range from 50 to 75. This implies that this study cannot note the efficacy of oleamide on individuals with more progressed cognitive decline or who are diagnosed as dementia. Also, this study cannot refer to its effect on individuals aged younger than 50 or older than 75. Further research is necessary to consider the effect of oleamide on the vast population. In addition, this intervention was solely targeted at the Japanese population. Thus, this study does not guarantee the effect of oleamide on the worldwide population. On the other hand, this clinical trial was conducted on both genders, a broad age range of middle-aged and elderly individuals, and those with relatively high cognitive levels. Therefore, this study suggests that continuous intake of oleamide could potentially improve cognitive function of elderly Japanese individuals in various conditions. If these results are universal, it could possibly benefit a broad spectrum of healthy elderly individuals in any ethnicity who are experiencing subjective cognitive decline. Additional research is warranted to understand the mechanism of action of oleamide on cognitive ability and to clarify its generalizability.

There are two potential future applications for oleamide functions. The initial step involves conducting other clinical studies to evaluate the effectiveness of MCW and oleamide on cognitive functions, particularly working memory and short-term memory. This new study will allow us to better understand the efficacy of MCW and oleamide on human cognitive function. This idea could be achieved by choosing an evaluation index that aligns more accurately with the participants’ characteristics and the specific cognitive functions we aim to study. Another option is to reexamine the efficacy of oleamide, focusing primarily on sleep function. This study showed significant improvements in sleep quality in both the MCW and oleamide groups when compared to the baseline; however, these improvements were not significantly different from that in the placebo group. Future studies on humans with sleep disorders could be conducted to gather evidence and clarify the beneficial effects of oleamide on sleep.

The current study revealed that the continuous intake of 60 μg of oleamide and MCW, which contains the same amount of oleamide, significantly improved the MPI score and both immediate and delayed free recall scores compared to placebo. This was observed after 12 weeks of intervention by elderly Japanese participants who were aware of their cognitive decline. Furthermore, oleamide significantly elevated the serum BDNF level. Taken together, these findings suggest that the continuous consumption of oleamide could help preserve or improve working and short-term memory in elderly Japanese individuals experiencing cognitive decline. In an aging society, strategies to prevent cognitive decline are crucial. Oleamide and foods containing it may aid in this endeavor. Moreover, MCW and oleamide have demonstrated the potential to improve sleep quality. This is the first clinical trial to report the efficacy of oleamide and oleamide-rich foods on cognitive function and sleep. Future studies should focus on providing more clinical evidence and understanding the underlying mechanism.

## Data availability statement

The raw data supporting the conclusions of this article will be made available by the authors, without undue reservation.

## Ethics statement

The studies involving humans were approved by Yoga Allergy Clinic’s Institutional Review Board. The studies were conducted in accordance with the local legislation and institutional requirements. The participants provided their written informed consent to participate in this study.

## Author contributions

MS: Writing – review & editing, Investigation, Methodology, Resources, Validation. CO: Conceptualization, Investigation, Methodology, Resources, Validation, Writing – review & editing. KN: Conceptualization, Investigation, Methodology, Project administration, Resources, Writing – review & editing. HTa: Data curation, Investigation, Writing – review & editing. HTo: Data curation, Formal analysis, Validation, Visualization, Writing – original draft. KF: Conceptualization, Funding acquisition, Supervision, Writing – review & editing.
