# Supplementary material for: Milk-based culture of Penicillium camemberti and its component oleamide affect cognitive function in healthy elderly Japanese individuals: a multi-arm randomized, double-blind, placebo-controlled study
Source: Front Nutr. 2024 Mar 27;11:1357920. doi: 10.3389/fnut.2024.1357920 (PMC11004446; doi:10.3389/fnut.2024.1357920)
Supplement: Supplementary file 1 [file Table_1.DOCX]

Supplementary Material

Milk-Based Culture of *Penicillium Camemberti* and Its Component Oleamide Enhance Cognitive Function in Healthy Elderly Japanese Individuals: A Multi-arm Randomized, Double-Blind, Placebo-Controlled Study

Mayuki Sasaki, Chisato Oba, Kentaro Nakamura, Hiroki Takeo, Hidemasa Toya, and Keisuke Furuichi

*** Correspondence:** Kentaro Nakamura: kentarou.nakamura@meiji.com

# Supplementary Tables

**S1 Table.** Vital and Physical Measurements.

|  | **Group** | **Baseline** | | | | | | | | | |  | **Week-12** | | | | | | | | | |  | **Δ** | | | | | | | | | |
| --- | --- | --- | --- | --- | --- | --- | --- | --- | --- | --- | --- | --- | --- | --- | --- | --- | --- | --- | --- | --- | --- | --- | --- | --- | --- | --- | --- | --- | --- | --- | --- | --- | --- |
|  |  | **N** | **Mean ± SD** | | | **95% CI** | | | | | **P-value** |  | **N** | **Mean ± SD** | | | **95% CI** | | | | | **P-value** |  | **N** | **Mean ± SD** | | | **95% CI** | | | | | **P-value** |
| Systolic Blood Pressure (mmHg) | Oleamide | 20 | 120.4 | ± | 14.8 | ( | -11.2 | – | 10.5 | ) | 0.996 |  | 20 | 122.6 | ± | 16.7 | ( | -10.2 | – | 10.8 | ) | 0.997 |  | 20 | 2.3 | ± | 10.8 | ( | -7.9 | – | 8.3 | ) | 0.997 |
|  | MCW | 20 | 118.5 | ± | 14.2 | ( | -13.1 | – | 8.7 | ) | 0.859 |  | 20 | 119.3 | ± | 13.4 | ( | -13.5 | – | 7.4 | ) | 0.730 |  | 20 | 0.8 | ± | 8.0 | ( | -9.4 | – | 6.8 | ) | 0.906 |
|  | Placebo | 20 | 120.7 | ± | 16.4 |  |  |  |  |  |  |  | 19 | 122.3 | ± | 12.7 |  |  |  |  |  |  |  | 19 | 2.1 | ± | 13.9 |  |  |  |  |  |  |
| Diastolic Blood Pressure (mmHg) | Oleamide | 20 | 75.5 | ± | 11.5 | ( | -10.7 | – | 3.9 | ) | 0.473 |  | 20 | 78.4 | ± | 11.3 | ( | -5.4 | – | 7.5 | ) | 0.908 |  | 20 | 2.9 | ± | 5.4 | ( | -1.5 | – | 8.2 | ) | 0.215 |
|  | MCW | 20 | 75.2 | ± | 8.1 | ( | -11.0 | – | 3.7 | ) | 0.426 |  | 20 | 76.2 | ± | 8.2 | ( | -7.6 | – | 5.3 | ) | 0.894 |  | 20 | 1.0 | ± | 6.5 | ( | -3.4 | – | 6.3 | ) | 0.729 |
|  | Placebo | 20 | 78.9 | ± | 10.8 |  |  |  |  |  |  |  | 19 | 77.3 | ± | 6.3 |  |  |  |  |  |  |  | 19 | -0.4 | ± | 8.0 |  |  |  |  |  |  |
| Pulse rate (BPM) | Oleamide | 20 | 78.1 | ± | 8.8 | ( | -5.1 | – | 6.2 | ) | 0.964 |  | 20 | 76.3 | ± | 11.2 | ( | -4.0 | – | 10.8 | ) | 0.474 |  | 20 | -1.8 | ± | 10.6 | ( | -3.1 | – | 8.5 | ) | 0.461 |
|  | MCW | 20 | 71.6 | ± | 8.6 | ( | -11.5 | – | -0.3 | ) | 0.038* |  | 20 | 72.1 | ± | 11.0 | ( | -8.1 | – | 6.6 | ) | 0.962 |  | 20 | 0.5 | ± | 5.7 | ( | -0.8 | – | 10.8 | ) | 0.096 |
|  | Placebo | 20 | 77.5 | ± | 5.8 |  |  |  |  |  |  |  | 19 | 72.8 | ± | 7.9 |  |  |  |  |  |  |  | 19 | -4.5 | ± | 6.7 | ( |  |  |  |  |  |
| Body Weight (kg) | Oleamide | 20 | 60.3 | ± | 10.4 | ( | -9.1 | – | 8.2 | ) | 0.990 |  | 20 | 60.4 | ± | 9.9 | ( | -8.1 | – | 9.1 | ) | 0.986 |  | 20 | 0.1 | ± | 1.2 | ( | -0.8 | – | 0.8 | ) | 0.999 |
|  | MCW | 20 | 60.7 | ± | 12.4 | ( | -8.7 | – | 8.6 | ) | 0.999 |  | 20 | 60.8 | ± | 12.5 | ( | -7.7 | – | 9.5 | ) | 0.958 |  | 20 | 0.1 | ± | 0.9 | ( | -0.8 | – | 0.8 | ) | 0.995 |
|  | Placebo | 20 | 60.7 | ± | 13.2 |  |  |  |  |  |  |  | 19 | 59.9 | ± | 12.9 |  |  |  |  |  |  |  | 19 | 0.1 | ± | 1.1 | ( |  |  |  |  |  |
| BMI (kg/m^2^) | Oleamide | 20 | 22.6 | ± | 2.9 | ( | -2.4 | – | 2.0 | ) | 0.971 |  | 20 | 22.7 | ± | 2.7 | ( | -2.0 | – | 2.3 | ) | 0.982 |  | 20 | 0.1 | ± | 0.4 | ( | -0.3 | – | 0.3 | ) | 0.966 |
|  | MCW | 20 | 22.6 | ± | 3.3 | ( | -2.4 | – | 2.0 | ) | 0.973 |  | 20 | 22.7 | ± | 3.3 | ( | -2.0 | – | 2.3 | ) | 0.988 |  | 20 | 0.0 | ± | 0.4 | ( | -0.3 | – | 0.3 | ) | 0.997 |
|  | Placebo | 20 | 22.8 | ± | 3.1 |  |  |  |  |  |  |  | 19 | 22.5 | ± | 2.8 |  |  |  |  |  |  |  | 19 | 0.0 | ± | 0.4 |  |  |  |  |  |  |

MCW: Milk-Based Culture of White Mold, BMI: Body Mass Index, CI: Confidence Interval. Data are presented as mean ± standard deviation (SD). * p <0.05, analyzed by Dunnett's test (vs. placebo).

**S2 Table.** Blood Biochemical Analysis.

|  | **Group** | **Baseline** | | | | | | | | | |  | **Week-12** | | | | | | | | | |  | **Δ** | | | | | | | | | |
| --- | --- | --- | --- | --- | --- | --- | --- | --- | --- | --- | --- | --- | --- | --- | --- | --- | --- | --- | --- | --- | --- | --- | --- | --- | --- | --- | --- | --- | --- | --- | --- | --- | --- |
|  |  | **N** | **Mean ± SD** | | | **95% CI** | | | | | **P-value** |  | **N** | **Mean ± SD** | | | **95% CI** | | | | | **P-value** |  | **N** | **Mean ± SD** | | | **95% CI** | | | | | **P-value** |
| TG (mg/dL) | Oleamide | 20 | 113.4 | ± | 59.3 | ( | -27.8 | – | 71.4 | ) | 0.508 |  | 20 | 101.7 | ± | 44.0 | ( | -12.6 | – | 54.3 | ) | 0.276 |  | 20 | -11.7 | ± | 32.7 | ( | -32.8 | – | 27.3 | ) | 0.968 |
|  | MCW | 20 | 108.5 | ± | 88.7 | ( | -32.8 | – | 66.5 | ) | 0.660 |  | 20 | 91.4 | ± | 47.5 | ( | -22.9 | – | 44.0 | ) | 0.697 |  | 20 | -17.1 | ± | 60.2 | ( | -38.2 | – | 22.0 | ) | 0.765 |
|  | Placebo | 20 | 91.6 | ± | 54.6 |  |  |  |  |  |  |  | 19 | 80.9 | ± | 46.6 |  |  |  |  |  |  |  | 19 | -8.9 | ± | 19.6 |  |  |  |  |  |  |
| T-Cho (mg/dL) | Oleamide | 20 | 227.7 | ± | 30.9 | ( | -10.4 | – | 34.9 | ) | 0.370 |  | 20 | 228.7 | ± | 31.5 | ( | -10.6 | – | 34.3 | ) | 0.384 |  | 20 | 1.0 | ± | 16.4 | ( | -13.5 | – | 12.1 | ) | 0.989 |
|  | MCW | 20 | 231.5 | ± | 30.6 | ( | -6.7 | – | 38.7 | ) | 0.199 |  | 20 | 231.9 | ± | 30.2 | ( | -7.3 | – | 37.5 | ) | 0.226 |  | 20 | 0.5 | ± | 18.3 | ( | -14.0 | – | 11.6 | ) | 0.968 |
|  | Placebo | 20 | 215.5 | ± | 33.2 |  |  |  |  |  |  |  | 19 | 216.8 | ± | 30.9 |  |  |  |  |  |  |  | 19 | 1.6 | ± | 18.1 |  |  |  |  |  |  |
| BUN (mg/dL) | Oleamide | 20 | 13.5 | ± | 2.5 | ( | -4.6 | – | -0.1 | ) | 0.039* |  | 20 | 13.4 | ± | 2.7 | ( | -3.5 | – | 1.2 | ) | 0.446 |  | 20 | 0.0 | ± | 2.4 | ( | -0.6 | – | 3.4 | ) | 0.217 |
|  | MCW | 20 | 13.8 | ± | 3.0 | ( | -4.2 | – | 0.3 | ) | 0.095 |  | 20 | 13.3 | ± | 3.5 | ( | -3.6 | – | 1.1 | ) | 0.368 |  | 20 | -0.6 | ± | 3.1 | ( | -1.2 | – | 2.9 | ) | 0.530 |
|  | Placebo | 20 | 15.8 | ± | 3.8 |  |  |  |  |  |  |  | 19 | 14.5 | ± | 3.5 |  |  |  |  |  |  |  | 19 | -1.4 | ± | 2.9 |  |  |  |  |  |  |
| T-Bil (mg/dL) | Oleamide | 20 | 0.7 | ± | 0.2 | ( | -0.3 | – | 0.1 | ) | 0.669 |  | 20 | 0.7 | ± | 0.2 | ( | -0.2 | – | 0.1 | ) | 0.783 |  | 20 | 0.0 | ± | 0.2 | ( | -0.1 | – | 0.2 | ) | 0.729 |
|  | MCW | 20 | 0.9 | ± | 0.4 | ( | -0.1 | – | 0.4 | ) | 0.325 |  | 20 | 0.8 | ± | 0.3 | ( | -0.1 | – | 0.2 | ) | 0.669 |  | 20 | -0.1 | ± | 0.2 | ( | -0.2 | – | 0.1 | ) | 0.573 |
|  | Placebo | 20 | 0.8 | ± | 0.3 |  |  |  |  |  |  |  | 19 | 0.7 | ± | 0.2 |  |  |  |  |  |  |  | 19 | -0.1 | ± | 0.2 |  |  |  |  |  |  |
| TP (g/dL) | Oleamide | 20 | 7.3 | ± | 0.5 | ( | -0.1 | – | 0.5 | ) | 0.237 |  | 20 | 7.3 | ± | 0.4 | ( | -0.1 | – | 0.5 | ) | 0.133 |  | 20 | 0.0 | ± | 0.3 | ( | -0.2 | – | 0.3 | ) | 0.896 |
|  | MCW | 20 | 7.3 | ± | 0.4 | ( | -0.1 | – | 0.5 | ) | 0.164 |  | 20 | 7.3 | ± | 0.3 | ( | 0.0 | – | 0.5 | ) | 0.088 |  | 20 | 0.0 | ± | 0.3 | ( | -0.2 | – | 0.3 | ) | 0.896 |
|  | Placebo | 20 | 7.1 | ± | 0.3 |  |  |  |  |  |  |  | 19 | 7.0 | ± | 0.4 |  |  |  |  |  |  |  | 19 | -0.1 | ± | 0.3 |  |  |  |  |  |  |
| Alb (g/dL) | Oleamide | 20 | 4.3 | ± | 0.3 | ( | -0.1 | – | 0.3 | ) | 0.382 |  | 20 | 4.3 | ± | 0.3 | ( | -0.2 | – | 0.2 | ) | 0.949 |  | 20 | 0.0 | ± | 0.2 | ( | -0.2 | – | 0.1 | ) | 0.418 |
|  | MCW | 20 | 4.4 | ± | 0.2 | ( | 0.0 | – | 0.4 | ) | 0.047* |  | 20 | 4.4 | ± | 0.2 | ( | -0.1 | – | 0.3 | ) | 0.279 |  | 20 | 0.0 | ± | 0.2 | ( | -0.2 | – | 0.1 | ) | 0.525 |
|  | Placebo | 20 | 4.2 | ± | 0.2 |  |  |  |  |  |  |  | 19 | 4.2 | ± | 0.3 |  |  |  |  |  |  |  | 19 | 0.0 | ± | 0.2 |  |  |  |  |  |  |
| γ-GTP (U/L) | Oleamide | 20 | 32.8 | ± | 33.1 | ( | -12.1 | – | 24.8 | ) | 0.651 |  | 20 | 27.4 | ± | 14.9 | ( | -11.6 | – | 12.9 | ) | 0.988 |  | 20 | -5.4 | ± | 22.2 | ( | -15.9 | – | 5.8 | ) | 0.470 |
|  | MCW | 20 | 25.7 | ± | 18.7 | ( | -19.2 | – | 17.7 | ) | 0.993 |  | 20 | 23.7 | ± | 12.5 | ( | -15.2 | – | 9.2 | ) | 0.802 |  | 20 | -2.0 | ± | 9.5 | ( | -12.4 | – | 9.2 | ) | 0.922 |
|  | Placebo | 20 | 26.4 | ± | 23.1 |  |  |  |  |  |  |  | 19 | 26.7 | ± | 22.0 |  |  |  |  |  |  |  | 19 | -0.4 | ± | 8.8 |  |  |  |  |  |  |
| AST (U/L) | Oleamide | 20 | 22.1 | ± | 8.0 | ( | -4.6 | – | 6.2 | ) | 0.921 |  | 20 | 20.4 | ± | 3.4 | ( | -6.8 | – | 1.2 | ) | 0.202 |  | 20 | -1.7 | ± | 5.5 | ( | -7.0 | – | 0.0 | ) | 0.052 |
|  | MCW | 20 | 23.6 | ± | 8.8 | ( | -3.1 | – | 7.7 | ) | 0.530 |  | 20 | 24.3 | ± | 5.8 | ( | -2.9 | – | 5.1 | ) | 0.741 |  | 20 | 0.8 | ± | 5.3 | ( | -4.6 | – | 2.5 | ) | 0.726 |
|  | Placebo | 20 | 21.3 | ± | 5.6 |  |  |  |  |  |  |  | 19 | 23.2 | ± | 6.8 |  |  |  |  |  |  |  | 19 | 1.8 | ± | 3.5 |  |  |  |  |  |  |
| ALT (U/L) | Oleamide | 20 | 19.8 | ± | 14.2 | ( | -7.2 | – | 11.0 | ) | 0.849 |  | 20 | 17.9 | ± | 5.7 | ( | -8.4 | – | 5.6 | ) | 0.857 |  | 20 | -2.0 | ± | 11.3 | ( | -9.4 | – | 3.2 | ) | 0.427 |
|  | MCW | 20 | 22.1 | ± | 14.4 | ( | -4.9 | – | 13.2 | ) | 0.482 |  | 20 | 23.0 | ± | 13.6 | ( | -3.3 | – | 10.7 | ) | 0.384 |  | 20 | 0.9 | ± | 9.1 | ( | -6.5 | – | 6.0 | ) | 0.993 |
|  | Placebo | 20 | 17.9 | ± | 8.4 |  |  |  |  |  |  |  | 19 | 19.3 | ± | 7.5 |  |  |  |  |  |  |  | 19 | 1.2 | ± | 3.1 |  |  |  |  |  |  |
| Cr (mg/dL) | Oleamide | 20 | 0.8 | ± | 0.2 | ( | -0.1 | – | 0.2 | ) | 0.643 |  | 20 | 0.8 | ± | 0.2 | ( | -0.1 | – | 0.1 | ) | 0.796 |  | 20 | 0.0 | ± | 0.1 | ( | 0.0 | – | 0.0 | ) | 0.976 |
|  | MCW | 20 | 0.8 | ± | 0.1 | ( | -0.1 | – | 0.1 | ) | 0.965 |  | 20 | 0.7 | ± | 0.1 | ( | -0.1 | – | 0.1 | ) | 0.919 |  | 20 | 0.0 | ± | 0.0 | ( | 0.0 | – | 0.1 | ) | 0.534 |
|  | Placebo | 20 | 0.7 | ± | 0.2 |  |  |  |  |  |  |  | 19 | 0.7 | ± | 0.2 |  |  |  |  |  |  |  | 19 | 0.0 | ± | 0.1 |  |  |  |  |  |  |
| UA (mg/dL) | Oleamide | 20 | 5.7 | ± | 1.0 | ( | -0.4 | – | 1.3 | ) | 0.383 |  | 20 | 5.3 | ± | 0.8 | ( | -0.6 | – | 1.2 | ) | 0.642 |  | 20 | -0.4 | ± | 0.8 | ( | -0.6 | – | 0.3 | ) | 0.697 |
|  | MCW | 20 | 5.1 | ± | 1.3 | ( | -1.0 | – | 0.7 | ) | 0.940 |  | 20 | 5.0 | ± | 1.4 | ( | -0.9 | – | 0.9 | ) | 0.998 |  | 20 | -0.1 | ± | 0.6 | ( | -0.4 | – | 0.6 | ) | 0.792 |
|  | Placebo | 20 | 5.3 | ± | 1.2 |  |  |  |  |  |  |  | 19 | 5.0 | ± | 1.4 |  |  |  |  |  |  |  | 19 | -0.2 | ± | 0.5 |  |  |  |  |  |  |
| LDL-Cho (mg/dL) | Oleamide | 20 | 133.9 | ± | 32.8 | ( | -14.4 | – | 31.9 | ) | 0.600 |  | 20 | 133.6 | ± | 30.8 | ( | -12.2 | – | 32.3 | ) | 0.487 |  | 20 | -0.3 | ± | 20.5 | ( | -13.9 | – | 13.6 | ) | 0.999 |
|  | MCW | 20 | 135.9 | ± | 32.3 | ( | -12.4 | – | 33.9 | ) | 0.471 |  | 20 | 137.0 | ± | 28.9 | ( | -8.8 | – | 35.7 | ) | 0.293 |  | 20 | 1.1 | ± | 19.6 | ( | -12.5 | – | 15.0 | ) | 0.968 |
|  | Placebo | 20 | 125.2 | ± | 31.6 |  |  |  |  |  |  |  | 19 | 123.5 | ± | 32.2 |  |  |  |  |  |  |  | 19 | -0.2 | ± | 16.5 |  |  |  |  |  |  |
| Glucose (mg/dL) | Oleamide | 20 | 91.1 | ± | 8.3 | ( | -7.1 | – | 5.7 | ) | 0.956 |  | 20 | 91.3 | ± | 7.5 | ( | -8.0 | – | 4.2 | ) | 0.701 |  | 20 | 0.3 | ± | 5.0 | ( | -6.4 | – | 2.6 | ) | 0.535 |
|  | MCW | 20 | 90.5 | ± | 11.3 | ( | -7.7 | – | 5.2 | ) | 0.868 |  | 20 | 92.6 | ± | 10.4 | ( | -6.7 | – | 5.5 | ) | 0.963 |  | 20 | 2.1 | ± | 7.5 | ( | -4.6 | – | 4.5 | ) | 0.999 |
|  | Placebo | 20 | 91.8 | ± | 6.6 |  |  |  |  |  |  |  | 19 | 93.2 | ± | 6.8 |  |  |  |  |  |  |  | 19 | 2.2 | ± | 6.0 |  |  |  |  |  |  |
| HDL-Cho (mg/dL) | Oleamide | 20 | 66.6 | ± | 15.6 | ( | -13.6 | – | 10.5 | ) | 0.939 |  | 20 | 67.7 | ± | 20.4 | ( | -18.6 | – | 7.9 | ) | 0.560 |  | 20 | 1.1 | ± | 8.3 | ( | -7.7 | – | 1.8 | ) | 0.283 |
|  | MCW | 20 | 68.8 | ± | 18.6 | ( | -11.4 | – | 12.7 | ) | 0.989 |  | 20 | 71.0 | ± | 18.4 | ( | -15.3 | – | 11.2 | ) | 0.913 |  | 20 | 2.2 | ± | 6.1 | ( | -6.6 | – | 2.9 | ) | 0.587 |
|  | Placebo | 20 | 68.2 | ± | 16.0 |  |  |  |  |  |  |  | 19 | 73.0 | ± | 15.5 |  |  |  |  |  |  |  | 19 | 4.0 | ± | 4.8 |  |  |  |  |  |  |
| ALP (U/L) | Oleamide | 20 | 68.1 | ± | 18.4 | ( | -18.4 | – | 10.3 | ) | 0.748 |  | 20 | 70.0 | ± | 17.4 | ( | -11.5 | – | 14.5 | ) | 0.948 |  | 20 | 1.9 | ± | 10.2 | ( | -3.3 | – | 9.4 | ) | 0.442 |
|  | MCW | 20 | 66.9 | ± | 18.0 | ( | -19.7 | – | 9.1 | ) | 0.615 |  | 20 | 66.8 | ± | 18.2 | ( | -14.7 | – | 11.3 | ) | 0.935 |  | 20 | -0.1 | ± | 6.3 | ( | -5.3 | – | 7.4 | ) | 0.899 |
|  | Placebo | 20 | 72.2 | ± | 23.3 |  |  |  |  |  |  |  | 19 | 68.5 | ± | 18.0 |  |  |  |  |  |  |  | 19 | -1.2 | ± | 9.1 |  |  |  |  |  |  |
| LDH (U/L) | Oleamide | 20 | 187.0 | ± | 33.5 | ( | -28.9 | – | 11.2 | ) | 0.505 |  | 20 | 184.2 | ± | 25.5 | ( | -34.1 | – | 4.8 | ) | 0.161 |  | 20 | -2.8 | ± | 21.9 | ( | -21.8 | – | 7.2 | ) | 0.417 |
|  | MCW | 20 | 189.9 | ± | 22.9 | ( | -25.9 | – | 14.1 | ) | 0.729 |  | 20 | 190.5 | ± | 19.4 | ( | -27.8 | – | 11.1 | ) | 0.518 |  | 20 | 0.6 | ± | 21.1 | ( | -18.4 | – | 10.5 | ) | 0.762 |
|  | Placebo | 20 | 195.8 | ± | 26.4 |  |  |  |  |  |  |  | 19 | 198.9 | ± | 33.8 |  |  |  |  |  |  |  | 19 | 4.5 | ± | 16.2 |  |  |  |  |  |  |
| HbA1c (%) | Oleamide | 20 | 5.5 | ± | 0.2 | ( | -0.1 | – | 0.3 | ) | 0.630 |  | 20 | 5.4 | ± | 0.3 | ( | -0.2 | – | 0.3 | ) | 0.782 |  | 20 | 0.0 | ± | 0.1 | ( | -0.2 | – | 0.1 | ) | 0.665 |
|  | MCW | 20 | 5.5 | ± | 0.4 | ( | -0.1 | – | 0.3 | ) | 0.665 |  | 20 | 5.4 | ± | 0.3 | ( | -0.2 | – | 0.3 | ) | 0.748 |  | 20 | 0.0 | ± | 0.2 | ( | -0.1 | – | 0.1 | ) | 0.791 |
|  | Placebo | 20 | 5.4 | ± | 0.3 |  |  |  |  |  |  |  | 19 | 5.4 | ± | 0.4 |  |  |  |  |  |  |  | 19 | 0.0 | ± | 0.2 |  |  |  |  |  |  |

MCW: Milk-Based Culture of White Mold, TG: Triglyceride, T-Cho: Total Cholesterol, BUN: Blood Urea Nitrogen, T-Bil: Total Bilirubin, TP: Total Protein, Alb: Albumin, γ-GTP: γ-Glutamyl Transpeptidase, AST: Aspartate Aminotransferase, ALT: Alanine Aminotransferase, Cr: Creatinine, UA: Uric Acid, LDL-Cho: LDL-Cholesterol, HDL-Cho: HDL-Cholesterol, ALP: Alkaline Phosphatase, LDH: Lactate Dehydrogenase, HbA1c: Hemoglobin A1c, CI: Confidence Interval. Data are presented as mean ± standard deviation (SD). * p <0.05, analyzed by Dunnett's test (vs. placebo).

**S3 Table.** Hematological Analysis.

|  | **Group** | **Baseline** | | | | | | | | | |  | **Week-12** | | | | | | | | | |  | **Δ** | | | | | | | | | |
| --- | --- | --- | --- | --- | --- | --- | --- | --- | --- | --- | --- | --- | --- | --- | --- | --- | --- | --- | --- | --- | --- | --- | --- | --- | --- | --- | --- | --- | --- | --- | --- | --- | --- |
|  |  | **N** | **Mean ± SD** | | | **95% CI** | | | | | **P-value** |  | **N** | **Mean ± SD** | | | **95%CI** | | | | | **P-value** |  | **N** | **Mean ± SD** | | | **95% CI** | | | | | **P-value** |
| WBC (/μL) | Oleamide | 20 | 5065.0 | ± | 796.2 | ( | -775.8 | – | 1215.8 | ) | 0.834 |  | 20 | 5085.0 | ± | 1064.4 | ( | -375.4 | – | 1187.5 | ) | 0.395 |  | 20 | 20.0 | ± | 762.7 | ( | -549.9 | – | 758.3 | ) | 0.908 |
|  | MCW | 20 | 5235.0 | ± | 1996.4 | ( | -605.8 | – | 1385.8 | ) | 0.579 |  | 20 | 4965.0 | ± | 1302.7 | ( | -495.4 | – | 1067.5 | ) | 0.617 |  | 20 | -270.0 | ± | 1047.4 | ( | -839.9 | – | 468.3 | ) | 0.743 |
|  | Placebo | 20 | 4845.0 | ± | 1078.2 |  |  |  |  |  |  |  | 19 | 4678.9 | ± | 782.9 |  |  |  |  |  |  |  | 19 | -84.2 | ± | 866.2 |  |  |  |  |  |  |
| RBC (×10⁴/μL) | Oleamide | 20 | 456.0 | ± | 29.7 | ( | -13.2 | – | 40.4 | ) | 0.412 |  | 20 | 457.3 | ± | 31.9 | ( | -5.1 | – | 52.0 | ) | 0.119 |  | 20 | 1.3 | ± | 20.4 | ( | -8.7 | – | 22.3 | ) | 0.509 |
|  | MCW | 20 | 457.7 | ± | 36.7 | ( | -11.5 | – | 42.1 | ) | 0.333 |  | 20 | 463.0 | ± | 42.5 | ( | 0.7 | – | 57.7 | ) | 0.043* |  | 20 | 5.3 | ± | 23.3 | ( | -4.7 | – | 26.3 | ) | 0.203 |
|  | Placebo | 20 | 442.4 | ± | 44.2 |  |  |  |  |  |  |  | 19 | 433.8 | ± | 42.7 |  |  |  |  |  |  |  | 19 | -5.5 | ± | 20.0 |  |  |  |  |  |  |
| Hb (g/dL) | Oleamide | 20 | 13.9 | ± | 1.1 | ( | -0.4 | – | 1.1 | ) | 0.520 |  | 20 | 13.9 | ± | 1.1 | ( | -0.3 | – | 1.2 | ) | 0.344 |  | 20 | -0.1 | ± | 0.7 | ( | -0.4 | – | 0.5 | ) | 0.953 |
|  | MCW | 20 | 14.2 | ± | 1.0 | ( | -0.2 | – | 1.3 | ) | 0.182 |  | 20 | 14.3 | ± | 1.0 | ( | 0.1 | – | 1.6 | ) | 0.029* |  | 20 | 0.1 | ± | 0.6 | ( | -0.2 | – | 0.7 | ) | 0.386 |
|  | Placebo | 20 | 13.6 | ± | 1.2 |  |  |  |  |  |  |  | 19 | 13.5 | ± | 1.2 |  |  |  |  |  |  |  | 19 | -0.1 | ± | 0.6 |  |  |  |  |  |  |
| Ht (%) | Oleamide | 20 | 43.4 | ± | 3.4 | ( | -1.4 | – | 3.3 | ) | 0.548 |  | 20 | 42.9 | ± | 2.7 | ( | -0.4 | – | 3.9 | ) | 0.125 |  | 20 | -0.6 | ± | 2.2 | ( | -0.9 | – | 2.1 | ) | 0.555 |
|  | MCW | 20 | 44.1 | ± | 2.9 | ( | -0.7 | – | 4.0 | ) | 0.202 |  | 20 | 44.1 | ± | 2.9 | ( | 0.8 | – | 5.1 | ) | 0.005* |  | 20 | 0.0 | ± | 2.0 | ( | -0.4 | – | 2.6 | ) | 0.161 |
|  | Placebo | 20 | 42.5 | ± | 3.5 |  |  |  |  |  |  |  | 19 | 41.1 | ± | 3.2 |  |  |  |  |  |  |  | 19 | -1.2 | ± | 2.0 |  |  |  |  |  |  |
| MCV (fL) | Oleamide | 20 | 95.3 | ± | 5.4 | ( | -4.3 | – | 2.5 | ) | 0.768 |  | 20 | 93.9 | ± | 4.0 | ( | -4.3 | – | 1.9 | ) | 0.584 |  | 20 | -1.4 | ± | 2.3 | ( | -1.6 | – | 1.5 | ) | 0.997 |
|  | MCW | 20 | 96.6 | ± | 4.5 | ( | -3.0 | – | 3.7 | ) | 0.957 |  | 20 | 95.5 | ± | 3.9 | ( | -2.7 | – | 3.5 | ) | 0.946 |  | 20 | -1.1 | ± | 1.9 | ( | -1.3 | – | 1.8 | ) | 0.885 |
|  | Placebo | 20 | 96.2 | ± | 4.1 |  |  |  |  |  |  |  | 19 | 95.1 | ± | 4.9 |  |  |  |  |  |  |  | 19 | -1.4 | ± | 2.2 |  |  |  |  |  |  |
| MCH (pg) | Oleamide | 20 | 30.6 | ± | 2.0 | ( | -1.5 | – | 1.1 | ) | 0.882 |  | 20 | 30.4 | ± | 1.8 | ( | -2.0 | – | 0.6 | ) | 0.345 |  | 20 | -0.2 | ± | 0.5 | ( | -0.8 | – | 0.1 | ) | 0.113 |
|  | MCW | 20 | 31.0 | ± | 1.5 | ( | -1.1 | – | 1.5 | ) | 0.938 |  | 20 | 31.0 | ± | 1.5 | ( | -1.4 | – | 1.1 | ) | 0.944 |  | 20 | 0.0 | ± | 0.7 | ( | -0.7 | – | 0.2 | ) | 0.413 |
|  | Placebo | 20 | 30.8 | ± | 1.9 |  |  |  |  |  |  |  | 19 | 31.1 | ± | 2.0 |  |  |  |  |  |  |  | 19 | 0.2 | ± | 0.6 |  |  |  |  |  |  |
| MCHC (%) | Oleamide | 20 | 32.1 | ± | 0.8 | ( | -0.5 | – | 0.6 | ) | 0.977 |  | 20 | 32.4 | ± | 0.7 | ( | -0.8 | – | 0.2 | ) | 0.231 |  | 20 | 0.3 | ± | 0.8 | ( | -0.9 | – | 0.2 | ) | 0.209 |
|  | MCW | 20 | 32.1 | ± | 0.7 | ( | -0.5 | – | 0.7 | ) | 0.931 |  | 20 | 32.4 | ± | 0.6 | ( | -0.8 | – | 0.3 | ) | 0.420 |  | 20 | 0.3 | ± | 0.7 | ( | -0.8 | – | 0.2 | ) | 0.301 |
|  | Placebo | 20 | 32.0 | ± | 0.9 |  |  |  |  |  |  |  | 19 | 32.7 | ± | 0.8 |  |  |  |  |  |  |  | 19 | 0.7 | ± | 0.7 |  |  |  |  |  |  |
| PLT (×10⁴/μL) | Oleamide | 20 | 26.5 | ± | 5.5 | ( | -1.9 | – | 5.2 | ) | 0.476 |  | 20 | 27.5 | ± | 5.2 | ( | -0.9 | – | 6.4 | ) | 0.170 |  | 20 | 1.0 | ± | 2.0 | ( | -0.5 | – | 2.7 | ) | 0.226 |
|  | MCW | 20 | 23.3 | ± | 4.5 | ( | -5.1 | – | 2.0 | ) | 0.500 |  | 20 | 23.7 | ± | 4.8 | ( | -4.8 | – | 2.5 | ) | 0.703 |  | 20 | 0.4 | ± | 2.1 | ( | -1.2 | – | 2.0 | ) | 0.785 |
|  | Placebo | 20 | 24.8 | ± | 4.7 |  |  |  |  |  |  |  | 19 | 24.8 | ± | 5.2 |  |  |  |  |  |  |  | 19 | 0.0 | ± | 2.4 |  |  |  |  |  |  |

MCW: Milk-Based Culture of White Mold, WBC: White Blood Cells, RBC: Red Blood Cells, Hb: Hemoglobin, Ht: Hematocrit, MCV: Mean Corpuscular Volume, MCH: Mean Corpuscular Hemoglobin, MCHC: Mean Corpuscular Hemoglobin Concentration, PLT: Platelets, CI: Confidence Interval. Data are presented as mean ± standard deviation (SD). * p <0.05, analyzed by Dunnett's test (vs. placebo).

**S4 Table.** Urinalysis (1).

|  | **Group** | **Baseline** | | | | | | | | | |  | **Week-12** | | | | | | | | | |  | **Δ** | | | | | | | | | |
| --- | --- | --- | --- | --- | --- | --- | --- | --- | --- | --- | --- | --- | --- | --- | --- | --- | --- | --- | --- | --- | --- | --- | --- | --- | --- | --- | --- | --- | --- | --- | --- | --- | --- |
|  |  | **N** | **Mean ± SD** | | | **95% CI** | | | | | **P-value** |  | **N** | **Mean ± SD** | | | **95% CI** | | | | | **P-value** |  | **N** | **Mean ± SD** | | | **95% CI** | | | | | **P-value** |
| pH | Oleamide | 20 | 5.80 | ± | 0.55 | ( | -0.50 | – | 0.45 | ) | 0.989 |  | 20 | 5.98 | ± | 0.68 | ( | -0.73 | – | 0.26 | ) | 0.460 |  | 20 | 0.18 | ± | 0.63 | ( | -0.74 | – | 0.36 | ) | 0.641 |
|  | MCW | 20 | 6.15 | ± | 0.86 | ( | -0.15 | – | 0.80 | ) | 0.222 |  | 20 | 6.35 | ± | 0.78 | ( | -0.36 | – | 0.64 | ) | 0.750 |  | 20 | 0.20 | ± | 0.91 | ( | -0.72 | – | 0.38 | ) | 0.711 |
|  | Placebo | 20 | 5.83 | ± | 0.54 |  |  |  |  |  |  |  | 19 | 6.21 | ± | 0.58 |  |  |  |  |  |  |  | 19 | 0.37 | ± | 0.70 |  |  |  |  |  |  |
| S.G. | Oleamide | 20 | 1.0180 | ± | 0.0064 | ( | -0.0059 | – | 0.0039 | ) | 0.858 |  | 20 | 1.0154 | ± | 0.0068 | ( | -0.0041 | – | 0.0054 | ) | 0.928 |  | 20 | -0.0026 | ± | 0.0064 | ( | -0.0031 | – | 0.0055 | ) | 0.752 |
|  | MCW | 20 | 1.0190 | ± | 0.0064 | ( | -0.0049 | – | 0.0050 | ) | 0.999 |  | 20 | 1.0153 | ± | 0.0061 | ( | -0.0041 | – | 0.0054 | ) | 0.938 |  | 20 | -0.0037 | ± | 0.0055 | ( | -0.0042 | – | 0.0044 | ) | 0.998 |
|  | Placebo | 20 | 1.0190 | ± | 0.0077 |  |  |  |  |  |  |  | 19 | 1.0147 | ± | 0.0067 |  |  |  |  |  |  |  | 19 | -0.0038 | ± | 0.0058 |  |  |  |  |  |  |

MCW: Milk-Based Culture of White Mold, pH: Potential of Hydrogen, S.G.: Specific Gravity, CI: Confidence Interval. Data are presented as mean ± standard deviation (SD). Analysis was conducted by Dunnett's test (vs. placebo).

**S5 Table.** Urinalysis (2).

|  | **Group** | **Baseline** | | | | | | |  | **Week-12** | | | | | | |
| --- | --- | --- | --- | --- | --- | --- | --- | --- | --- | --- | --- | --- | --- | --- | --- | --- |
|  |  | **N** | **-** | **±** | **1＋** | **2＋** | **3＋** | **4＋** |  | **N** | **-** | **±** | **1＋** | **2＋** | **3＋** | **4＋** |
| Protein | Oleamide | 20 | 16 | 3 | 1 | 0 | 0 | 0 |  | 20 | 18 | 0 | 2 | 0 | 0 | 0 |
|  | MCW | 20 | 18 | 2 | 0 | 0 | 0 | 0 |  | 20 | 20 | 0 | 0 | 0 | 0 | 0 |
|  | Placebo | 20 | 17 | 3 | 0 | 0 | 0 | 0 |  | 19 | 19 | 0 | 0 | 0 | 0 | 0 |
| Glucose | Oleamide | 20 | 19 | 1 | 0 | 0 | 0 | 0 |  | 20 | 20 | 0 | 0 | 0 | 0 | 0 |
|  | MCW | 20 | 20 | 0 | 0 | 0 | 0 | 0 |  | 20 | 20 | 0 | 0 | 0 | 0 | 0 |
|  | Placebo | 20 | 20 | 0 | 0 | 0 | 0 | 0 |  | 19 | 19 | 0 | 0 | 0 | 0 | 0 |
| Urobilinogen | Oleamide | 20 | - | 20 | 0 | 0 | 0 | 0 |  | 20 | - | 20 | 0 | 0 | 0 | 0 |
|  | MCW | 20 | - | 20 | 0 | 0 | 0 | 0 |  | 20 | - | 20 | 0 | 0 | 0 | 0 |
|  | Placebo | 20 | - | 20 | 0 | 0 | 0 | 0 |  | 19 | - | 19 | 0 | 0 | 0 | 0 |
| Occult Blood | Oleamide | 20 | 19 | 0 | 0 | 1 | 0 | - |  | 20 | 16 | 2 | 0 | 1 | 1 | - |
|  | MCW | 20 | 18 | 2 | 0 | 0 | 0 | - |  | 20 | 18 | 1 | 0 | 1 | 0 | - |
|  | Placebo | 20 | 20 | 0 | 0 | 0 | 0 | - |  | 19 | 18 | 1 | 0 | 0 | 0 | - |
| Bilirubin | Oleamide | 20 | 20 | 0 | 0 | 0 | 0 | - |  | 20 | 20 | 0 | 0 | 0 | 0 | - |
|  | MCW | 20 | 20 | 0 | 0 | 0 | 0 | - |  | 20 | 20 | 0 | 0 | 0 | 0 | - |
|  | Placebo | 20 | 20 | 0 | 0 | 0 | 0 | - |  | 19 | 19 | 0 | 0 | 0 | 0 | - |
| Keton body | Oleamide | 20 | 20 | 0 | 0 | 0 | 0 | - |  | 20 | 20 | 0 | 0 | 0 | 0 | - |
|  | MCW | 20 | 20 | 0 | 0 | 0 | 0 | - |  | 20 | 20 | 0 | 0 | 0 | 0 | - |
|  | Placebo | 20 | 20 | 0 | 0 | 0 | 0 | - |  | 19 | 19 | 0 | 0 | 0 | 0 | - |

MCW: Milk-Based Culture of White Mold, CI: Confidence Interval. Data are presented as variation in the number of participants in the reference values. Oleamide and MCW groups showed no significance compared to placebo group (analyzed by Steel’s test).
